# Stress hyperglycemia and risk of adverse outcomes in patients with acute ischemic stroke: a systematic review and dose–response meta–analysis of cohort studies

**DOI:** 10.3389/fneur.2023.1219863

**Published:** 2023-11-23

**Authors:** Yong-Wei Huang, Zong-Ping Li, Xiao-Shuang Yin

**Affiliations:** ^1^Department of Neurosurgery, Mianyang Central Hospital, School of Medicine, University of Electronic Science and Technology of China, Mianyang, Sichuan, China; ^2^Department of Immunology, Mianyang Central Hospital, School of Medicine, University of Electronic Science and Technology of China, Mianyang, Sichuan, China

**Keywords:** stress hyperglycemia, acute ischemic stroke, stress hyperglycemia ratio, dose-response meta-analysis, clinical outcome

## Abstract

**Background:**

Stroke represents a prominent global health issue, exhibiting the third highest incidence of disability and a significant burden on both healthcare and the economy. Stress hyperglycemia, an acute reaction of the hypothalamic-pituitary-adrenal axis and the sympathetic nervous system, leading to adverse outcomes and mortality. Several previous studies have indicated that stress hyperglycemia, as evaluated by the stress hyperglycemia ratio (SHR), significantly increases the risk of adverse outcomes and mortality in stroke patients. However, there is a lack of further investigation into the influence of dynamic changes in stress hyperglycemia on the clinical outcomes of acute ischemic stroke (AIS) patients. Consequently, we performed a meticulous analysis, considering dose-response relationships from existing studies, to ascertain the correlation between dynamic changes in stress hyperglycemia and the susceptibility to adverse outcomes in patients with AIS.

**Methods:**

This investigation was prospectively registered in PROSPERO and adhered to the PRISMA guidelines. A comprehensive search was performed across English and Chinese databases. A two-sided random-effects model was employed to consolidate the odds ratios (ORs) of the highest vs. lowest categories of SHR. Restricted cubic spline (RCS) models were employed to estimate potential non-linear trends between SHR and the risk of adverse outcomes in AIS patients. Egger's test was utilized to assess publication bias. Heterogeneity was evaluated using Cochran's *Q*-test. The Newcastle-Ottawa Scale (NOS) tool was employed to evaluate the risk of bias of the included studies.

**Results:**

The final analysis incorporated a total of thirteen studies, which were published between 2019 and 2023, encompassing a participant cohort of 184,179 individuals. The SHR exhibited a significant association with the risk of various adverse outcomes. Specifically, a higher SHR was correlated with a 2.64-fold increased risk of 3-month poor functional outcomes (OR: 2.64, 95% CI 2.05–3.41, *I*^2^ = 52.3%, *P* < 0.001), a 3.11-fold increased risk of 3-month mortality (OR: 3.11, 95% CI 2.10–4.59, *I*^2^ = 38.6%, *P* < 0.001), a 2.80-fold increased risk of 1-year mortality (OR: 2.80, 95% CI 1.81–4.31, *I*^2^ = 88%, *P* < 0.001), a 3.90-fold increased risk of intracerebral hemorrhage (ICH) and 4.57-fold increased risk of symptomatic ICH (sICH) (ICH-OR: 3.90, 95% CI 1.52–10.02, *I*^2^ = 84.3%, *P* = 0.005; sICH-OR: 4.57, 95% CI 2.05–10.10, *I*^2^ = 47.3%, *P* < 0.001), a 1.73-fold increased risk of neurological deficits (OR: 1.73, 95 CI 1.44–2.08, *I*^2^ = 0%, *P* < 0.001), and a 2.84-fold increased risk of stroke recurrence (OR: 2.84, 95 CI 1.48–5.45, *I*^2^ = 50.3%, *P* = 0.002). It is noteworthy that, except for hemorrhagic transformation (HT) and stroke recurrence, the remaining adverse outcomes exhibited a “J-shaped” non-linear dose-response relationship.

**Conclusion:**

In summary, our findings collectively suggest that increased exposure to elevated SHR is robustly linked to a heightened risk of adverse outcomes and mortality in individuals with AIS, exhibiting a non-linear dose-response relationship. These results underscore the significance of SHR as a predictive factor for stroke prognosis. Therefore, further investigations are warranted to explore the role of SHR in relation to adverse outcomes in stroke patients from diverse ethnic populations. Furthermore, there is a need to explore the potential benefits of stress hyperglycemia control in alleviating the physical health burdens associated with AIS. Maintaining a lower SHR level may potentially reduce the risk of adverse stroke outcomes.

**Systematic review registration:**

https://www.crd.york.ac.uk/prospero/, identifier: CRD42023424852.

## 1 Introduction

Stroke is a major global health issue, being the second leading cause of death and third leading cause of disability worldwide (1). In 2019, there were 12.2 million new stroke cases, resulting in 6.55 million deaths and a loss of 143 million disability-adjusted life years (1). Acute ischemic stroke (AIS), which occurs from blocked cerebral arteries, represents about 87% of all strokes (2). The financial impact of stroke treatment and aftercare is also significant. A meta-analysis showed that the worldwide cost of stroke, covering both direct and indirect healthcare expenses, varies between $1,809.51 and $325,108.84, placing a heavy burden on healthcare systems (3).

In AIS patients, hyperglycemia can be triggered by an acute stress response from the hypothalamic-pituitary-adrenal axis and the sympathetic nervous system (4). This hyperglycemia is linked to larger ischemic areas, worsened functional outcomes, and higher mortality (5). Surprisingly, about one-third of AIS patients show stress-induced hyperglycemia even without prior diabetes (6, 7). Current research emphasizes the importance of admission glucose levels, considering them a key modifiable factor for AIS outcomes (8–10). While some studies associate stress-induced hyperglycemia with prognosis, their clinical application can be limited by factors like diabetes control and diet (11–13). To overcome this, Roberts et al. (13) proposed the the concept of stress-hyperglycemia ratio (SHR). In the following studies, SHR was proved to be a potential prognostic indicator in critically ill, acute myocardial infarction, and AIS patients (14–17). This ratio compares stress-induced blood glucose to measures like HbA1c or estimated average glucose (EAG). Multiple formulas exist for calculating SHR, such as fasting glucose (mmol/L)/HbA1c (%) (18). The EAG formula, introduced by Nathan et al., is [(1.59 × HbA1c) – 2.59] (19). Another related metric, the glucose-to-HbA1c ratio (GAR), uses FBG (mg/dL)/HbA1c (%) (17). Regardless of these variations, SHRs provide insight into stress-induced hyperglycemia in AIS, potentially offering a better gauge of its clinical impact.

Our prior research has unequivocally demonstrated that a higher SHR is significantly associated with an elevated risk of adverse outcomes, mortality, early neurological deterioration (END), hemorrhagic transformation (HT), and infectious complications, regardless of diabetes history and treatment methods (20). Nonetheless, further exploration into the influence of dynamic changes in stress hyperglycemia on AIS outcomes is essential. Thus, it is imperative to investigate the dose-response relationship between stress hyperglycemia and the risk of adverse outcomes in AIS patients. Considering the substantial burden that stroke places on healthcare systems and the economy, our study seeks to provide invaluable insights for the primary prevention of stroke.

## 2 Methods

The current study adhered to the guidelines outlined by the Preferred Reporting Items for Systematic Reviews and Meta-Analyses (PRISMA) (21). Additionally, it was prospectively registered at the International Prospective Register of Systematic Reviews (PROSPERO) with the identifier CRD42023424852 (22). The study's compliance with the PRISMA 2020 checklist can be found in Supplementary Table 1. The objective of this systematic review and meta-analysis was to investigate the relationship between various levels of stress hyperglycemia and the risk of adverse outcomes in patients diagnosed with AIS.

### 2.1 Search strategy

A systematic search of eligible studies was conducted in several databases, including PubMed, Embase, the Cochrane Library, the China National Knowledge Infrastructure (CNKI), Wan Fang, and the Chinese VIP Information (VIP) database. The search encompassed the period from the inception of the databases up until the end of July 2022, with language restrictions set to English and Chinese. It should be noted that, based on our previous study (20) and the data extraction characteristics in dose-response meta-analysis, a re-evaluation of the full-text articles from the previous 16 included studies was performed, resulting in the identification of 7 studies that met the inclusion criteria for the dose-response meta-analysis. Subsequently, the same search strategy was manually applied to search PubMed and Chinese databases for relevant studies published after August 1, 2022. We searched the Chinese databases manually using the search term “应激性高血糖比值” 或 “应激性高血糖比率” 或 “stress hyperglycemia ratio” 或 “SHR”, and then screened the literature one by one. Additionally, a thorough review of topic-related editorials, perspectives, methodologies, and comments was conducted to identify potentially useful information. Supplementary literature was also manually investigated for other relevant studies. To ensure comprehensive coverage, the reference lists of retrieved articles were checked manually to avoid any omissions. Detailed information regarding the systematic search process can be found in Supplementary Table 2.

### 2.2 Study selection

Inclusion and exclusion criteria were applied to select the appropriate studies for analysis. The following criteria were used for inclusion: (a) studies had to be published in a peer-reviewed journal; (b) publication had to be in either English or Chinese languages; (c) the exposure of interest had to be different stress hyperglycemia levels assessed by SHR; (d) the study had to investigate adverse outcomes related to stroke; (e) the availability of odd ratios (ORs) or hazard ratios (HRs) with corresponding 95% confidence intervals (CIs) for different endpoints; (f) the reported results had to be adjusted for relevant covariates. On the other hand, studies were excluded if they were in the form of reviews, letters, commentaries, conference abstracts, or case reports, as they did not meet the criteria for inclusion in the analysis.

### 2.3 Data extraction and quality assessment

Two reviewers conducted the data extraction process independently, using a predefined form that included the following information: first author's name, publication year, country, age, sex, number of participants and cases for different endpoints, SHR levels, endpoints, duration of follow-up, and covariates adjusted in the multivariable analysis. If a study reported multiple adjustment models, only the model with the highest number of adjusted covariates was included.

Furthermore, the quality assessment of the included studies was performed using the Newcastle-Ottawa Scale (NOS) (23). The NOS evaluates the studies based on three aspects: selection, comparability, and outcome. Studies that received more than six stars were considered to be of high quality. Any discrepancies during the data extraction and quality assessment process were resolved through consultation with a third author to ensure consensus.

### 2.4 The definition of adverse endpoints

The 3-month poor functional outcome was assessed by modified Rankin Scale (mRS), and the standard of the scale can be found in the Supplementary Table 3. According to the European Cooperative Acute Stroke Study II criteria, early neurological deterioration (END) is defined as an increase of ≥4 points in the National Institutes of Health Stroke Scale (NIHSS) score within 24 h after vascular recanalization therapy. Symptomatic intracerebral hemorrhage (sICH) is defined as the presence of brain parenchymal bleeding on head CT scans within 7 days after intravascular treatment, accompanied by an increase of ≥4 points in the NIHSS score compared to the baseline. Based on the study of Merlino et al. (24), intracranial hemorrhage (ICH) and sICH were regarded as hemorrhagic transformation (HT). Stroke recurrence was defined as a new neurological deficit (new ischemic stroke, hemorrhagic stroke, and transient ischemic attack) or re-hospitalization. The primary outcome was 3-month poor functional outcome and secondary outcomes were mortality at 3 months and 1 year, END, HT (ICH and sICH), and stroke recurrence.

### 2.5 Statistical analysis

In this meta-analysis, the effect sizes were represented by ORs and 95% CIs. ORs and HRs were considered equivalent. If the reference category for the SHR was not the first tertile, the method described by Orsini was applied to convert the reference category to the first tertile (25). When studies reported separate effect sizes for diabetes and non-diabetes subgroups, each subgroup was treated as an independent study. To ensure consistency, SHR levels reported in mg/dl were converted to mmol/L by dividing the values by 18. For each included study, we extracted the SHR values for each category. If the highest category was open-ended, we estimated the mean value by multiplying the upper bound of the category by 1.5. Similarly, if the lowest category was open-ended, we estimated the mean value by dividing the lower bound of the category by 1.5. Studies that provided fewer than three categories of SHR levels or did not report the numbers of cases or participants in each category were excluded from the dose-response meta-analysis. To pool the ORs of the highest vs. lowest categories of SHR from different studies, we employed a two-stage random-effects model (26). Publication bias was assessed using Egger's test (27). We also utilized restricted cubic spline models with three knots to examine potential non-linear trends between SHR and the risk of adverse outcomes (25, 28), calculating non-linear *p*-values (29). Cochran's *Q*-test was used to assess heterogeneity among studies, with significance set at *P* = 0.1 or *I*^2^ > 50% (30, 31). All statistical analyses were performed using R version 4.1.2 and the “meta” R package. Statistical significance was defined as *P* < 0.05.

## 3 Results

### 3.1 Study characteristics

**Figure 1:**
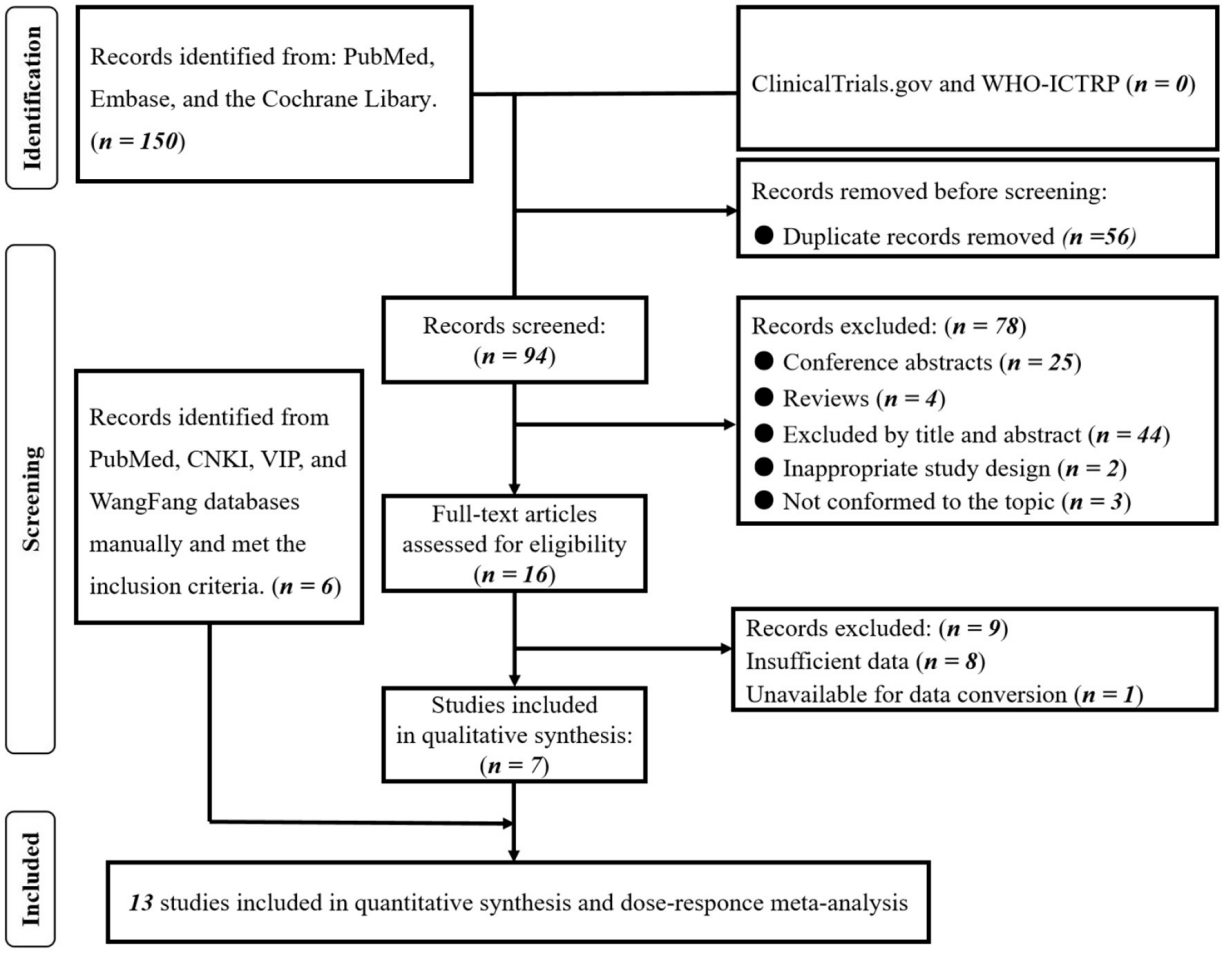
PRISMA flowchart of included studies. Based on our prior investigation (20) and the data extraction methods employed in the dose-response meta-analysis, we conducted a thorough reassessment of the complete texts of the 16 previously incorporated studies. Subsequently, 7 studies were identified as meeting the criteria for inclusion in the dose-response meta-analysis. Employing the identical search strategy, we manually searched the PubMed and Chinese databases, leading to the identification of an additional 6 studies that satisfied the inclusion criteria. Consequently, a total of 13 studies were ultimately included in this comprehensive dose-response meta-analysis.

The search strategy for this study was based on a previous study (20). Initially, 16 studies (10, 24, 32–45) underwent a full-text evaluation, out of which eight studies (32, 36–38, 40–42, 45) were excluded due to insufficient data, and one study by Wang et al. (33) was excluded because the reference group was the intermediate dose level group, making it impossible to calculate the OR of the highest vs. lowest dose level group using the algorithm provided by Orsini et al. (25), resulting in a total of seven (10, 24, 34, 35, 39, 43, 44) studies being included. Subsequently, a manual search was conducted in the mentioned electronic databases, leading to the inclusion of six additional studies (46–51) that met the criteria for the dose-response meta-analysis. In total, 13 studies (10, 24, 34, 35, 39, 43, 44, 46–51) were included in this systematic review and dose-response meta-analysis (Figure 1). These studies were published between 2019 and 2023 and involved a total of 184,179 participants (Table 1). Among the 13 studies, 10 were conducted in China and three in Italy. Four studies (35, 43, 47, 48) stratified the study population into diabetes and non-diabetes subgroups, with each subgroup treated as an independent study.

**Table 1:** Characteristics of studies included.

**References**	**Year**	**Country**	**Study design**	**Age (*y*)**	**Male (%)**	**Participants (*n*)**	**Cases (*n*)**	**SHR levels**	**Intervention**	**Endpoints**	**Clinical follow-up (*d*)**	**Adjustment**	**NOS score**
Merlino et al. (10)	2021	Italy	Chort	74.22 ± 3.65	53.38	414	189	Q1: 14.0 (13.3–14.4) Q2: 16.2 (15.6–16.6) Q3: 18.1 (17.5–18.9) Q4: 22.7 (21.5–25.9)	IVT	Poor outcome	90	Age, history of DM, baseline NIHSS score, pre-stroke mRS, and time from symptom onset to IVT	8
							57			Mortality	90		
							49			ICH	In-hospital	Age, history of DM, baseline NIHSS score, pre-stroke mRS, time from symptom onset to IVT, SBP > 180 mmHg.	
							24			sICH	In-hospital		
Merlino et al. (24)	2021	Italy	Chort	74.34 ± 3.57	49.02	204	122	Q1: 15.1 (13.6–15.7) Q2: 17.6 (16.9–18.1) Q3: 19.8 (18.9–20.8) Q4: 25.4 (23.1–28.4)	MT	Poor outcome	90	Age, history of DM, ASPECTS, baseline NIHSS score, pre-stroke mRS, time from symptom onset to MT, door-to-groin time, procedure duration.	8
							37			Mortality	90		
							53			ICH	In-hospital	Age, history of DM, ASPECTS, baseline NIHSS score, pre-stroke mRS, time from symptom onset to MT, door-to-groin time, procedure duration, SBP.	
							22			sICH	In-hospital		
Zhu et al. (34)	2019	China	Chort	61.70 ± 13.00	64.56	999	76	Q1: ≤ 0.82 Q2: 0.83–0.92 Q3: 0.93–1.06 Q4: ≥1.07	–	Mortality	365	Age, gender, NIHSS, stroke subtype, history of hypertension, hyperlipidemia, CHD, AF, smoking status, pulmonary infection, urinary infection during hospitalization, statins, thrombolytic, antiplatelet, antihypertensive, anticoagulation agents use during hospitalization.	9
							105			Stroke recurrence			
Li et al. (35)	2020	China	Chort	65.23 ± 2.89	62.80	3,488 in DM cohort	286	Q1: ≤ 15.30 Q2: 15.30–17.43 Q3: 17.43–20.16 Q4: >20.16	–	Mortality	365	Age, sex, current or previous smoking, medical histories of hypertension, DM, hypercholesterolemia, AF, transient ischemic stroke, ischemic stroke, MI, CHF, baseline NIHSS and mRS scores, baseline SBP and DBP, baseline levels of LDL-C, triglyceride and hsCRP.	9
						2,838 in DM cohort	514			Neurological deficit			
						5,134 in non-DM cohort	392			Mortality		The aforementioned variables except for the history of DM.	
						4,146 in non-DM cohort	675			Neurological deficit			
Cai et al. (39)	2022	China	Chort	65.70 ± 12.60	61.70	846	200	Q1: ≤ 0.81 Q2: 0.82–0.91 Q3: 0.92–1.06 Q4: ≥1.07	–	Poor outcome	90	Sex, age, NIHSS, type of stroke, AF, hypertension, DM, CHD, hyperlipemia, previous stroke, history of smoking, history of drinking, and infectious complications	8
						846	35			Mortality	90		
						743	85				365		
Merlino et al. (43)	2022	Italy	Chort	74.75 ± 3.91	53.89	130 in DM cohort	21	Q1: 15.1 (13.1–16) Q2: 19.2 (18–20.4) Q3: 24.5 (22.4–27.6)	IVT	Mortality	90	Age, AF, creatinine, CRP, total cholesterol, LDL-C, stroke due to small vessel disease, baseline NIHSS score.	8
							72			Poor outcome	90	Age, stroke due to cardioembolism, stroke due to small vessel disease, baseline NIHSS score, pre-stroke mRS.	
							18			sICH	In-hospital	Protein, total cholesterol, stroke due to small vessel disease, baseline NIHSS score	
						371 in non-DM cohort	35	Q1: 14.2 (13.6–14.9) Q2: 16.7 (16.1–17.1) Q3: 20.4 (18.8–22.5)		Mortality	90	Age, sex, AF, creatinine, CRP, use of antiplatelets at admission, baseline NIHSS score, pre-stroke mRS.	
							151			Poor outcome	90	Age, sex, AF, CRP, ASPECTS, stroke due to large arterial atherosclerosis, baseline NIHSS score, pre-stroke mRS.	
							17			sICH	In-hospital	Age, AF, creatinine, triglycerides, baseline NIHSS score, pre-stroke mRS.	
Mi et al. (44)	2022	China	Chort	66.20 ± 10.70	56.99	168,381	882	Q1: 0–0.90 Q2: 0.90–1.08 Q3: 1.08–1.32 Q4: >1.32	–	Mortality	365	Age, sex, BMI, NIHSS on admission, hypertension, AF, previous ischemic stroke, previous MI, SAH, antiplatelet, anticoagulation, lipid-lowering drug, smoking, alcohol, LDL-C, FBG, HbA1c, eGFR, HCY, SBP, DBP, reperfusion therapeutic.	9
Liu et al. (46)	2022	China	Chort	63.00 ± 12.00	68.07	592	356	Q1: < 1.07 Q2: 1.07–1.23 Q3: 1.23–1.47 Q4: ≥1.47	EVT	Poor outcome	90	Age, sex, hypertension, history of DM, TIA/ischemic stroke, CHD, AF, onset-to-admission time, baseline NIHSS score, ASTIN/SIR.	8
							37			sICH			
							67			Neurological deficit			
Zhang et al. (47)	2022	China	Chort	70.37 ± 2.81	63.90	1,484	561	Q1: ≤ 0.73 Q2: 0.74–0.83 Q3: 0.84–0.98 Q4: >0.98	IVT with Alteplase EVT Other	Poor outcome	90	Age, sex, smoking, hypertension, baseline NIHSS score, history of DM, TIA/ischemic stroke, CHD, AF, Creatinine, FBG, HbA1c.	8
Duan et al. (48)	2023	China	Chort	67.07 ± 11.75	56.94	230 in DM cohort	36	Q1: ≤ 0.986 Q2: 0.986–1.187 Q3: 1.187–1.497 Q4: >1.497	IVT	Mortality	30	The regression model was adjusted for all potential confounders.	8
							122			Poor outcome	90		
						346 in non-DM cohort	45	Q1: ≤ 0.93 Q2: 0.93–1.075 Q3: 1.075–1.331 Q4: >1.331		Mortality	30		
							147			Poor outcome	90		
Peng et al. (49)	2023	China	Chort	67.00 ± 2.93	56.46	542	85	Q1: ≤ 1.07 Q2: 1.08–1.29 Q3: ≥1.30	–	Mortality	30	Age, sex, smoking, hypertension, hyperlipidemia, baseline NIHSS score, occlusion site, stroke etiology, history of DM, baseline ASPECTS.	8
							39			ICH	In-hospital		
							165			sICH	In-hospital		
Wang et al. (50)	2023	China	Chort	61.30 ± 3.06	69.51	610	58	Q1: 0.7 ± 0.1 Q2: 0.9 ± 0.1 Q3: 1.2 ± 0.2	–	Stroke recurrence	In-hospital	Age, sex, systolic pressure, diastolic pressure, smoking, BMI, NIHSS, Stroke history, hyperlipoidemia, antiplatelet therapy, statins therapy	8
Zhang et al. (51)	2023	China	Chort	64.69 ± 13.56	64.95	408	51	Q1: 0.82 ± 0.11 Q2: 1.03 ± 0.05 Q3: 1.23 ± 0.07 Q4: 1.75 ± 0.49	MT	Mortality	90	Age, NIHSS score at admission, pre-stroke mRS and IV tPA administration.	8
							110			ICH	In-hospital	Age, NIHSS score at admission, pre-stroke mRS, hemoglobin, HCY, INR, systolic blood pressure, onset-to-reperfusion time, ASPECTS, and IV tPA administration.	
							21			sICH	In-hospital	IV tPA administration.	

CHD, coronary heart disease; NLR, neutrophil/lymphocyte ratio; NIHSS, National Institutes of Health Stroke Scale; sICH, symptomatic intracranial hemorrhage; ICH, intracranial hemorrhage; AF, atrial fibrillation; MI, myocardial infarction; CHF, congestive heart failure; mRS, modified Rankin Scale; CRP, C-reactive protein; IVT, intravenous thrombolysis; MT, mechanical thrombectomy; EVT, endovascular treatment; ASPECTS, Alberta Stroke Program Early CT Score; LDL, low-density lipoprotein; HCY, homocysteine; hs-CRP, high-sensitive C-reactive protein; INR, international normalized ratio; BMI, body mass index; SAH, subarachnoid hemorrhage; ASTIN/SIR, American Society of Interventional and Therapeutic Neuroradiology/Society of Interventional Radiology; IV tPA, intravenous tissue plasminogen activator; TIA, transient ischemia attack; FBG, fasting blood glucose; HbA1c, glycated hemoglobin; LDL-C, low-density lipoprotein cholesterol; eGFR, estimated glomerular filtration rate; SBP, systolic blood pressure; DBP, diastolic blood pressure; DM, diabetes mellitus.

### 3.2 Dose-response meta-analysis of SHR and risk of different adverse endpoints

#### 3.2.1 SHR and risk of poor functional outcomes at 3-months

**Figure 2:**
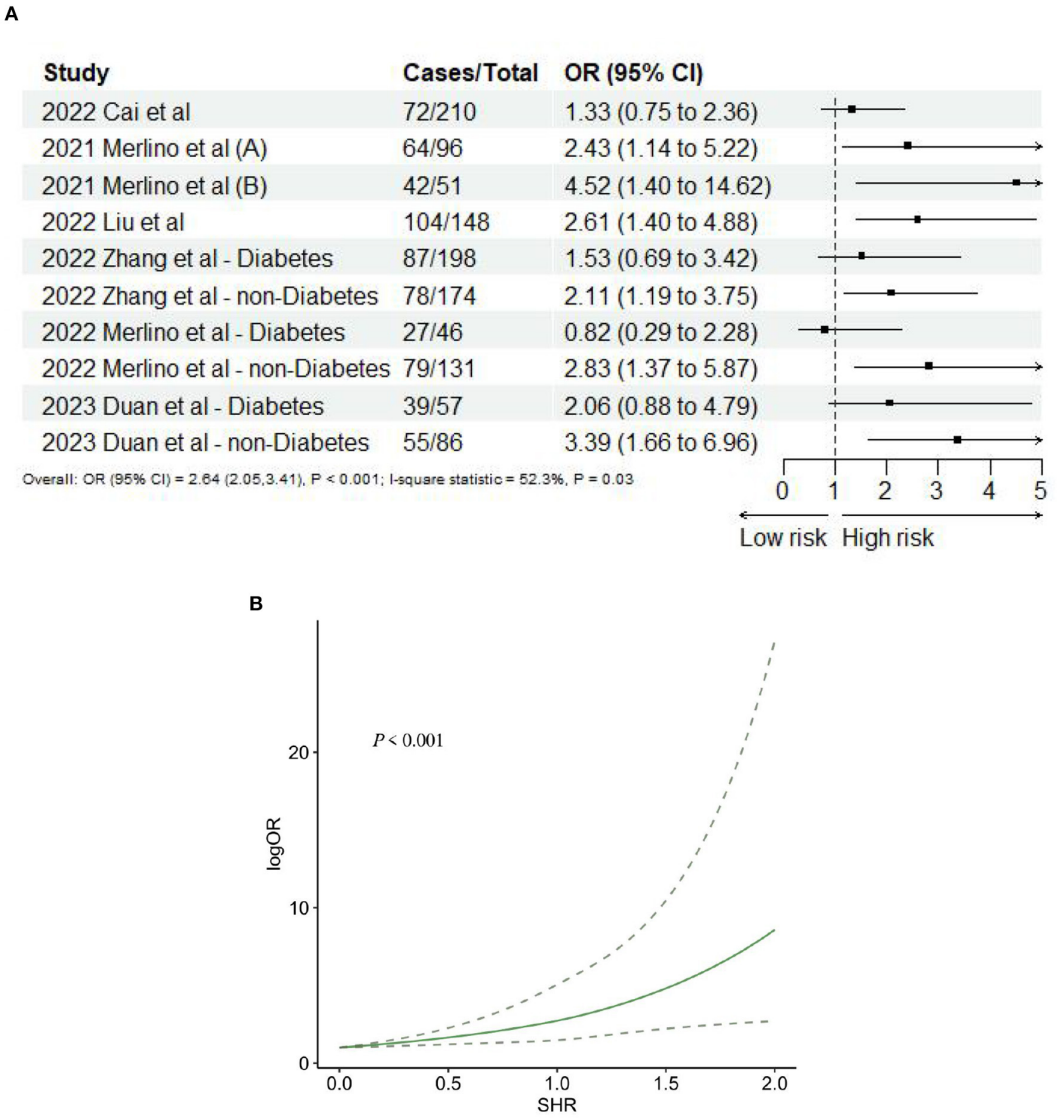
**(A)** Forest plot of the meta-analysis of highest vs. lowest SHR and risk of 3-months poor functional outcome. **(B)** The dose-response relationship between SHR and risk of 3-months poor functional outcome.

The summarized findings are presented in Table 2. Seven studies (10, 24, 39, 43, 46–48) provided data on 3-month poor functional outcome, and the sub-groups from three studies (43, 47, 48) were treated as independent entities. Consequently, a total of 10 records were included in the final analysis. The meta-analysis revealed a significant association between SHR and 3-month poor functional outcome (OR: 2.64, 95% CI: 2.05–3.41, *I*^2^ = 52.3%, *P* < 0.001, Figure 2A). The analysis further indicated that the risk of 3-month poor functional outcome remained relatively low when SHR was below 1.0. However, when SHR exceeded 1.0, the risk of poor functional outcome significantly increased, demonstrating a “J-shaped” non-linear relationship between 3-month poor functional outcome and SHR (*P* < 0.001; Figure 2B).

**Table 2:** Meta-analysis of different endpoints.

**Items**		**Results**		
		**Records**, ***n***	**Highest vs. lowest OR (95% CI)**	***P-*****value (heterogeneity**, *I*^2^ **and** ***P*** **for Cochran Q)**
3-month poor outcome		10	2.64 (2.05–3.41)	*P* < 0.001 (*I*^2^ = 52.3%, *P* = 0.03)
3-month mortality		9	3.11 (2.10–4.59)	*P* < 0.001 (*I*^2^ = 38.6%, *P* = 0.11)
1-year mortality		5	2.80 (1.81–4.31)	*P* < 0.001 (*I*^2^ = 88%, *P* < 0.001)
HT	ICH	4	3.90 (1.52–10.02)	*P* = 0.005 (*I*^2^ = 84.3%, *P* < 0.001)
	sICH	7	4.57 (2.05–10.10)	*P* < 0.001 (*I*^2^ = 47.7%, *P* = 0.07)
Neurological deficits		3	1.73 (1.44–2.08)	*P* < 0.001 (*I*^2^ = 0%, *P* = 0.55)
Stroke recurrence		2	2.84 (1.48–5.45)	*P* = 0.002 (*I*^2^ = 50.3%, *P* = 0.16)

#### 3.2.2 SHR and risk of 3-month and 1-year mortality

**Figure 3:**
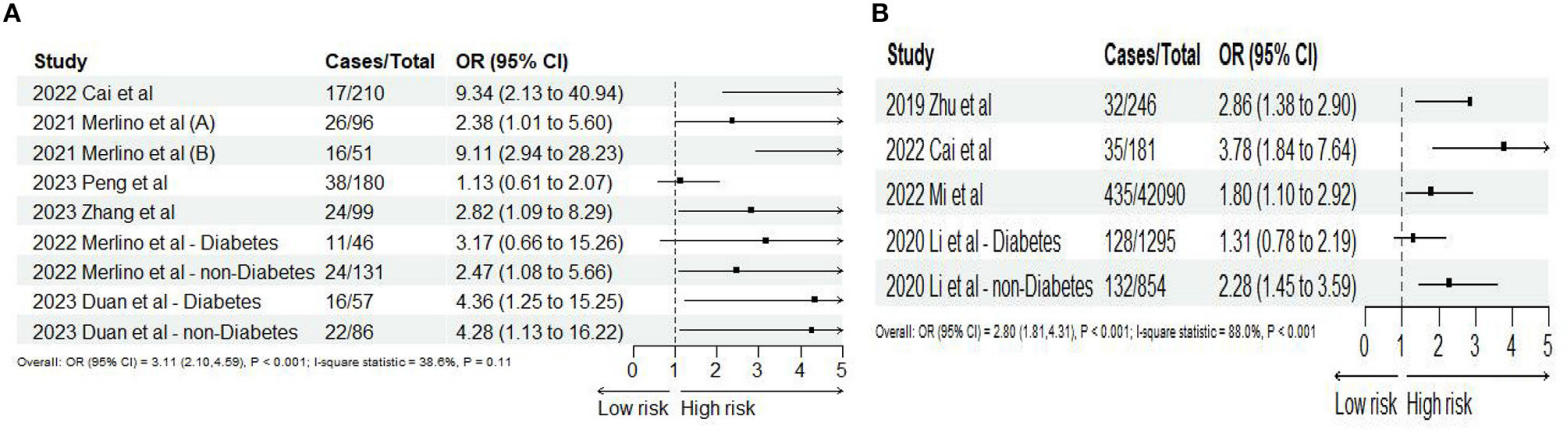
Forest plot of the meta-analysis of highest vs. lowest SHR and risk of **(A)** 3-months and **(B)** 1-year mortality.

**Figure 4:**
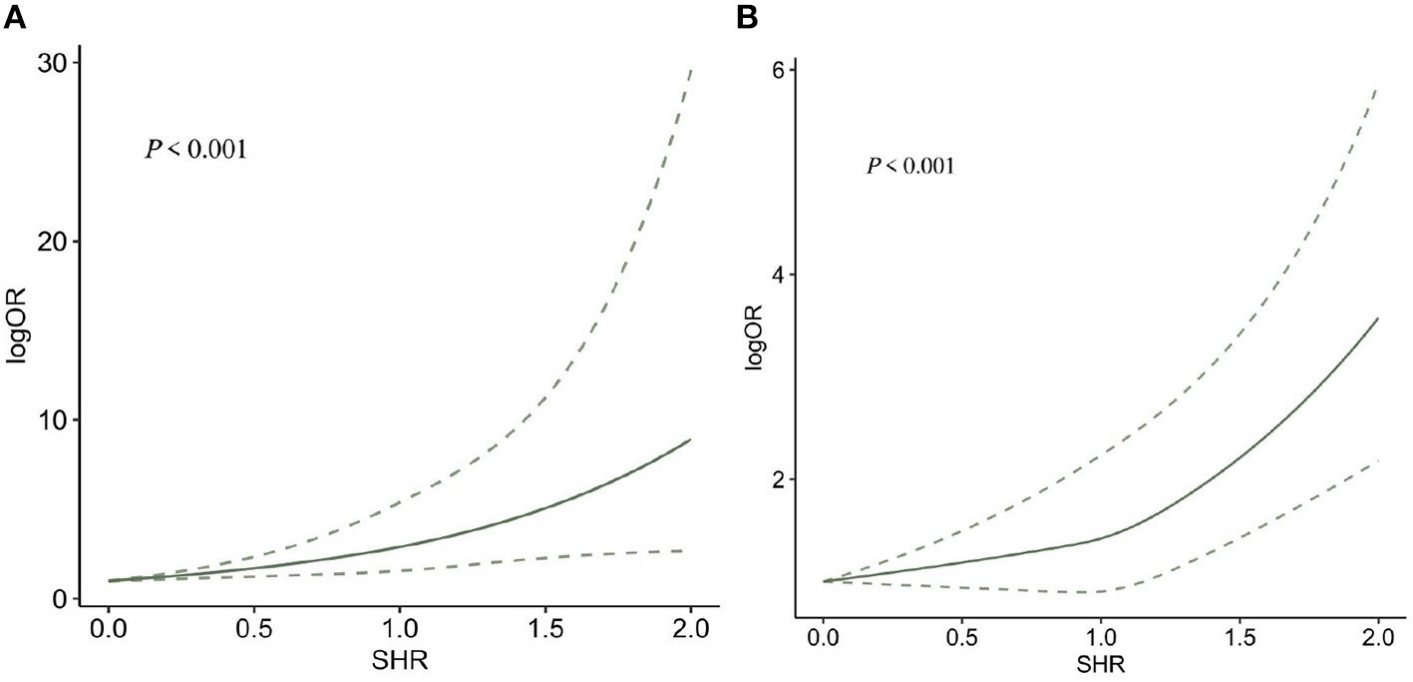
The dose-response relationship between SHR and risk of **(A)** 3-months and **(B)** 1-year mortality.

Seven (10, 24, 39, 43, 48, 49, 51) studies reported 3-month mortality, two (43, 48) of which divided the study population into diabetes and non-diabetes subgroups, and the sub-groups were regarded as independent studies. Hence, nine records were included in the final analysis. Our meta-analysis showed that SHR was significantly associated with 3-month mortality (OR: 3.11, 95% CI: 2.10–4.59, *I*^2^ = 38.6%, *P* < 0.001, Figure 3A). Similarly, four studies (34, 35, 39, 44) reported 1-year mortality, and the two sub-groups of one study (36) were regarded as independent studies. Lastly, five records were included in the final analysis. The meta-analysis showed that SHR was still significantly associated with 1-year mortality (OR: 2.80, 95% CI: 1.81–4.31, *I*^2^ = 88%, *P* < 0.001, Figure 3B). The risk of 3-month mortality was kept at a relative low point when SHR < 1.5. When SHR > 1.5, the risk of mortality significantly increases. For 1-year mortality, the risk of mortality was kept at a relative low point when SHR < 1.0. When SHR < 1.0, the risk of mortality significantly increases. Both the risk of 3-month and 1-year mortality and SHR satisfied the “J-shaped” non-linear relationship (both *P* < 0.001; Figures 4A, B).

#### 3.2.3 SHR and risk of HT

**Figure 5:**
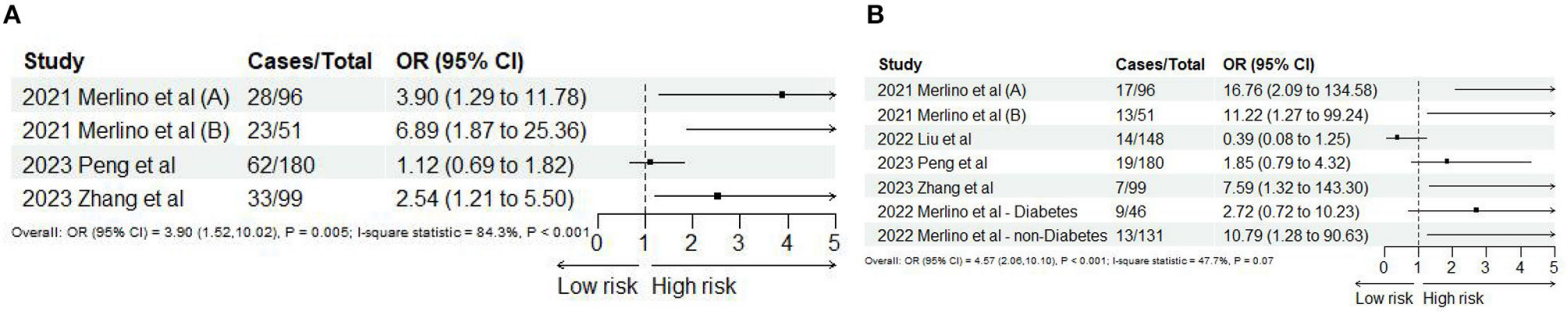
Forest plot of the meta-analysis of highest vs. lowest SHR and risk of **(A)** ICH and **(B)** sICH.

**Figure 6:**
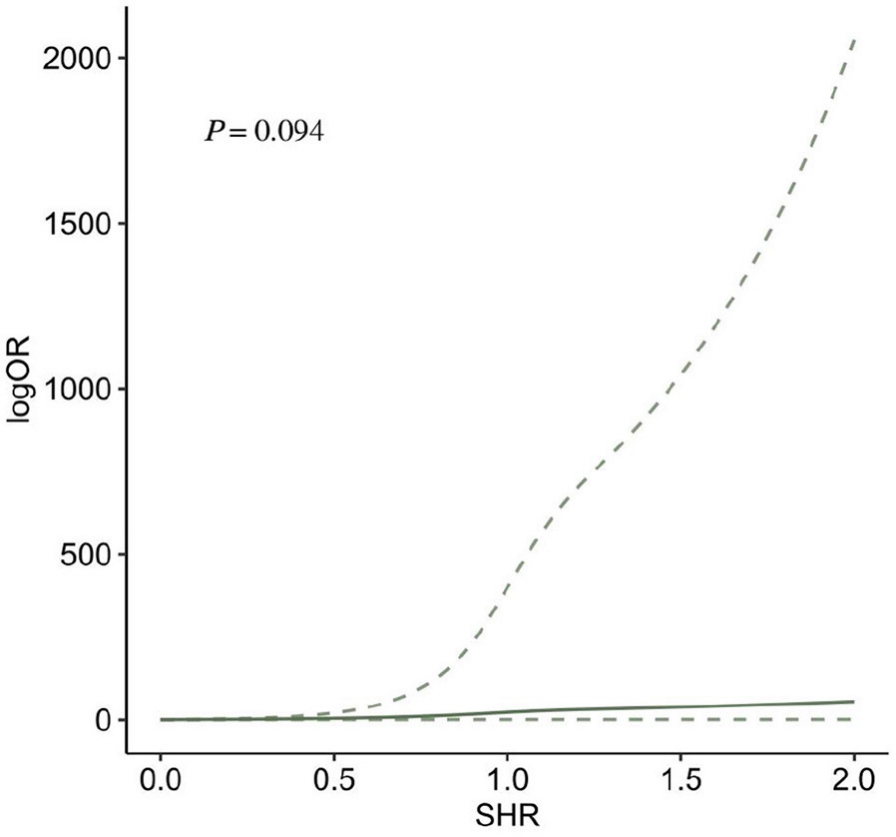
The dose-response relationship between SHR and risk of ICH.

Based on the study of Merlino et al. (24), ICH and sICH were regarded as HT. Six studies (10, 24, 45, 46, 49, 51) reported ICH and sICH, and 11 records satisfy the final analysis. The analysis showed that SHR was significantly associated with ICH (OR: 3.90, 95% CI: 1.52–10.02, *I*^2^ = 84.3%, *P* = 0.005, Figure 5A) and sICH (OR: 4.57, 95% CI: 2.05–10.10, *I*^2^ = 47.3%, *P* < 0.001, Figure 5B). When we conducted non-linear trend analysis by RCS model, because of the huge discrepancy in the included studies, the ORs of sICH were too low (close to 0) and the 95% CI was too wide, causing the algorithm to fail to balance convergence speed and accuracy so that it could not be applied. Besides, the risk of ICH and SHR was not satisfied with the non-linear relationship (*P* = 0.094; Figure 6).

#### 3.2.4 SHR and risk of END

**Figure 7:**
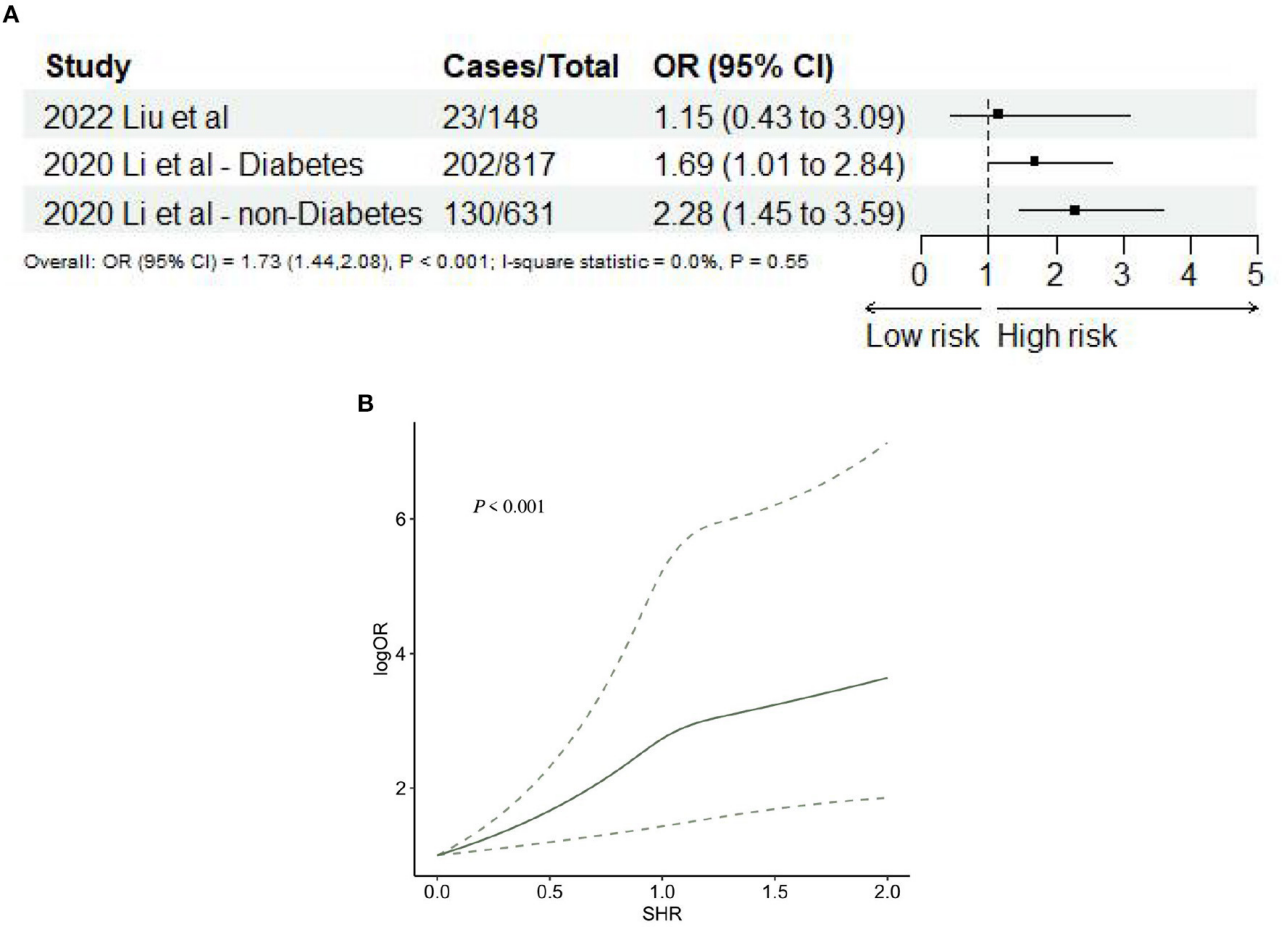
**(A)** Forest plot of the meta-analysis of highest vs. lowest SHR and risk of neurological deficit. **(B)** The dose-response relationship between SHR and risk of neurological deficits.

Two studies (35, 46), including three records, reported END after AIS. The meta-analysis showed that SHR was significantly associated with END (OR: 1.73, 95% CI: 1.44–2.08, *I*^2^ = 0%, *P* < 0.001, Figure 7A). The trend showed that the risk of END gradually increases when SHR increases. When SHR = 1.0, the risk of neurological deficit suddenly increases, and then the risk of END keeps increasing. SHR and the risk of END satisfied the non-linear relationship (*P* < 0.001; Figure 7B).

#### 3.2.5 SHR and risk of stroke recurrence

**Figure 8:**
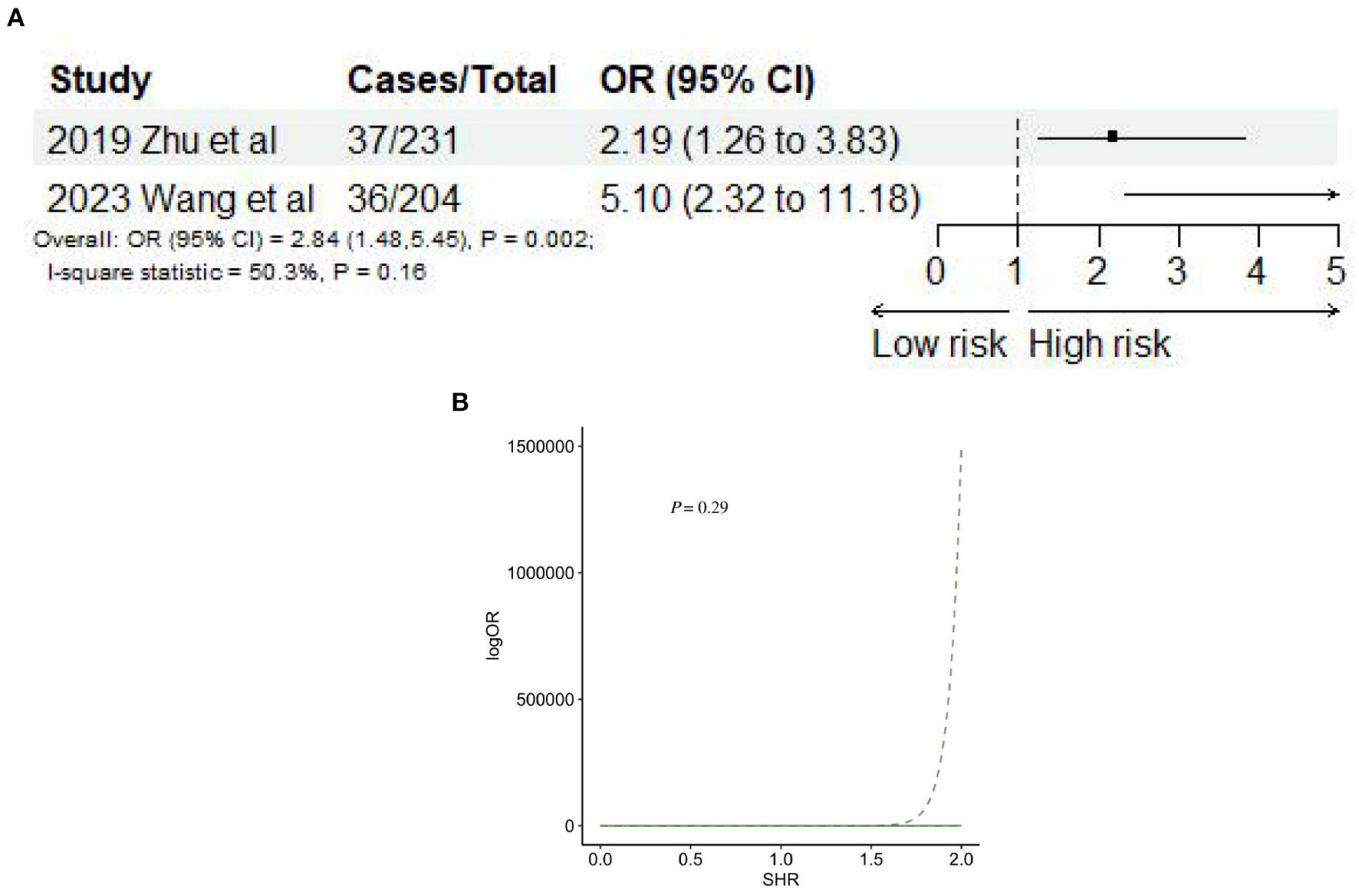
**(A)** Forest plot of the meta-analysis of highest vs. lowest SHR and risk of stroke recurrence. **(B)** The dose-response relationship between SHR and risk of stroke recurrence.

Two studies (34, 50) reported stroke recurrence after AIS. The meta-analysis showed that SHR was significantly associated with stroke recurrence (OR: 2.84, 95% CI: 1.48–5.45, *I*^2^ = 50.3%, *P* = 0.002, Figure 8A). Nevertheless, SHR and the risk of stroke recurrence did not satisfy the non-linear relationship (*P* = 0.29; Figure 8B).

### 3.3 Risk of bias assessment and publication bias assessment

The NOS tool was used to assess the methodological quality of the included studies, and they all received a median score of 8 stars, with an inter-quartile range ranging from 8 to 9 stars. This indicates that all studies were considered to be of high quality. The detailed assessment of the methodological quality for each study can be found in Supplementary Table 3. To evaluate the probability of publication bias, Egger's test was conducted, and the results indicated no evidence of publication bias (all *P*-values > 0.05).

## 4 Discussion

We first performed a dose-response meta-analysis on SHR's effects on AIS adverse outcomes in all individuals and specifically non-diabetics. RCS models further indicated a “J-shaped” non-linear relationship between SHR and adverse outcomes like poor functional outcomes at 3 months, mortality at 3 months and 1 year, END, HT, and stroke recurrence. However, this trend was not observed for HT and stroke recurrence. Notably, the worst outcomes were most linked to the highest SHR group, as studies often categorized SHR into tertiles or quartiles.

Shi et al. (52) found in their meta-analysis that blood glucose is crucial for stroke risk, with a non-linear relationship between FBG and stroke risk. As seen in Figure 3, the risk of 3-month mortality for diabetics is on par or higher than for non-diabetics, as shown by Duan et al. (48) and Merlino et al. (43). Yet, the 1-year mortality risk is lower for diabetics, as found by Li et al. (35). These mixed findings underline the complex relationship between diabetes and post-AIS outcomes. The initial 90 days post-AIS are vital for diabetic patients, aligning with previous studies (35, 43, 47, 48) that they may face more severe AIS effects or complications during this period. Hence, immediate optimal glycemic control post-stroke is essential. However, our results also suggest a lower 1-year mortality risk for diabetics, possibly due to long-term interventions like continuous blood glucose control and lifestyle changes. Further research is needed to understand these observations better.

Various equations, such as FGB (mmol/L)/HbA1c (%), have been commonly used to calculate the SHR to assess the relationship between glycemic control and stroke outcomes. However, it is crucial to acknowledge the potential limitations associated with this equation, particularly concerning comorbidities known to affect HbA1c levels, such as hemoglobinopathies or anemia. Comorbid conditions like hemoglobinopathies or anemia can significantly impact HbA1c measurements, rendering the use of the FBG/HbA1c equation less reliable in certain cases. These conditions can lead to variations in HbA1c levels that may not accurately reflect long-term glycemic control. Therefore, caution is necessary when interpreting SHR values in individuals with such comorbidities, as they may result in an overestimation or underestimation of the true association between glycemic control and stroke outcomes. Researchers and clinicians must be aware of these potential confounding factors and consider them when utilizing SHR as a metric to evaluate the relationship between diabetes management and stroke risk. Alternative methods or adjustments may be needed in specific patient populations to ensure a more accurate assessment of glycemic control in relation to stroke outcomes.

SHR provides a more precise measure of stress-induced hyperglycemia by adjusting for prior diabetes history and baseline blood glucose levels, thus better indicating disease prognosis (10, 53). Earlier studies have linked elevated GAR with worse outcomes in acute ischemic stroke patients receiving thrombolytic therapy (24). However, for broader understanding and application, Nathan proposed using EAG over HbA1c or glycated albumin (8, 34, 35). Although SHR offers a more logical and scientifically sound choice, research on its clinical relevance in AIS is limited, especially for those undergoing IVT and EVT (18, 33, 39).

The pathophysiological mechanisms underlying the association between SHR and stroke severity as well as prognosis in patients with acute ischemic stroke are still not fully elucidated. A particular study employed correlation analysis to shed light on this relationship, revealing that higher SHR levels are predominantly linked to increased stroke severity and heightened inflammatory responses upon admission (46). These findings align with a potential mechanistic framework through which SHR may impact stroke prognosis and potentially contribute to a self-perpetuating cycle. On one hand, the occurrence of a stroke represents a stress event that triggers the activation of the hypothalamic-pituitary-adrenal axis and the sympathetic nervous system (4, 54). Dysregulation of neuroendocrine hormones promotes glucagon secretion (55, 56), hinders insulin release, induces insulin resistance, stimulates liver glycogenolysis and gluconeogenesis (57), ultimately leading to elevated blood glucose levels. Moreover, as stroke severity escalates, so does the intensity of the stress response, consequently contributing to higher SHR levels. On the other hand, hyperglycemia has the potential to induce inflammation and the release of vasoconstrictive agents (57). Animal experiments have demonstrated that hyperglycemia can cause cerebral vasospasm through the NO pathway (58), induce platelet aggregation through superoxide production, activate the coagulation system, inhibit the fibrinolytic system, generate free radicals, and make patients more susceptible to ischemic injury (59). Hyperglycemia may also damage the blood-brain barrier, exacerbate cerebral edema, and cause more severe ischemic brain damage (60). Additionally, high blood glucose levels may lead to endothelial dysfunction and oxidative stress in stroke patients, resulting in reduced cerebral blood flow and impaired cerebral blood flow autoregulation, exacerbating ischemic injury (61–63). Finally, hyperglycemia may have direct neurotoxic effects on the ischemic penumbra, leading to neuronal damage (17, 58, 64) and worsening the stroke's severity. This cascade of reactions may culminate in a detrimental feedback loop that ultimately influences the clinical prognosis of stroke patients.

China, due to its population and rising risk factors, is witnessing a surge in AIS incidence. Multiple studies from China have detailed the relationship between stress hyperglycemia and AIS outcomes (32, 34, 35, 39, 44, 46–51). Stress hyperglycemia is consistently associated with increased mortality, functional impairment, and complications following AIS (10, 24, 34, 35, 39, 43, 44, 48, 49, 51). Zhang et al. (47) linked high SHR with poor 90-day outcomes in non-diabetic AIS patients. Interestingly, the impact of stress hyperglycemia varies based on the presence of diabetes. Non-diabetic patients with severe stress hyperglycemia face higher risks, while the same doesn't hold for diabetic patients, potentially due to chronic exposure to hyperglycemia (48). Regardless of diabetes status, SHR predicts stroke recurrence in AIS patients with intracranial atherosclerotic stenosis (50). The findings emphasize the need for personalized glucose control and diligent glycemic management after a stroke, especially considering its effects on treatment outcomes.

While Perez-Vega et al. (65) indeed demonstrated the prognostic significance of glucose levels at admission for patients undergoing MT for large-vessel occlusion, it's essential to consider the broader context of glucose monitoring and its relation to stress hyperglycemia. Blood glucose levels upon admission, though clinically simpler, offer a snapshot of the patient's current metabolic state. However, they might not provide a complete picture of the patient's physiological response to acute stress, which is where the SHR becomes particularly relevant. The SHR offers a more comprehensive insight into the patient's acute stress response. Stress hyperglycemia, or the acute elevation of blood glucose in response to physiological stressors, is different from chronic hyperglycemia. By using SHR, we can better differentiate between patients who have high glucose levels primarily due to their chronic metabolic state (e.g., uncontrolled diabetes) and those who are experiencing a significant stress response. Furthermore, while the timely adjustment of SHR by HbA1c level might be a concern due to the lag in HbA1c measurement, it's important to note that HbA1c represents an average glucose level over ~2–3 months. Hence, a recent HbA1c measurement (within the last month or so) would be sufficiently relevant for calculating SHR in the acute setting.

To the best of our current knowledge, this dose-response meta-analysis represents a pioneering effort in exploring the impact of dynamic fluctuations in stress hyperglycemia on susceptibility to adverse outcomes among individuals with AIS. Our findings unequivocally establish a positive association between the Stress-Hyperglycemia Ratio (SHR) and the risk of adverse outcomes in AIS patients. Furthermore, we have observed a distinct “J-shaped” non-linear dose-response pattern, indicating that elevated SHR is significantly correlated with an increased likelihood of 3-month poor functional outcomes, 3-month and 1-year mortality, as well as neurological deficits. These conclusions are in alignment with prior research, thereby deepening our understanding of the influence of dynamic changes in stress hyperglycemia on the risk of adverse outcomes in AIS patients. In summary, these investigations provide invaluable insights into the intricate relationship between stress hyperglycemia and AIS patient outcomes, underscoring the critical importance of implementing effective glycemic management strategies within this specific population. Further research endeavors are warranted to delve into the complex interplay between glucose control approaches and prognosis among stroke patients originating from diverse ethnic backgrounds and populations.

## 5 Limitation

Our study has several limitations that merit consideration. Firstly, a majority of the included studies possessed a retrospective cohort study design, which lacks the advantages of randomized controlled trials, notwithstanding their substantial sample sizes. Secondly, the predominant representation of studies conducted by Chinese researchers limits the generalizability of our findings to other ethnicities and countries. To gain a more comprehensive understanding of the relationship between stress hyperglycemia and adverse outcomes in AIS patients, it is imperative to include studies from diverse populations. Thirdly, the dose-response meta-analysis incorporated only two studies reporting stroke recurrence after AIS, highlighting the urgency for further research focusing on this intriguing theme. Fourthly, the reporting of adverse outcomes exhibited heterogeneity owing to variations in the duration of follow-up among the included studies, which could contribute to the overall heterogeneity observed in the analysis. Lastly, The substantial heterogeneity between stress hyperglycemia and the risk of 1-year mortality and ICH is high, and in principle, performing a subgroup analysis or meta-regression would indeed be an ideal approach to explore potential sources of this heterogeneity. However, due to the limited number of studies included in our analysis, it's not feasible to conduct a robust meta-regression. Insufficient studies could lead to unstable and overly sensitive results in a meta-regression. Nevertheless, despite these limitations, the results of our meta-analysis still offer valuable insights for clinicians when making treatment decisions for AIS patients.

## 6 Conclusion

In summary, our findings collectively suggest that increased exposure to elevated SHR is robustly linked to a heightened risk of adverse outcomes and mortality in individuals with AIS, exhibiting a non-linear dose-response relationship. These results underscore the significance of SHR as a predictive factor for stroke prognosis. Therefore, further investigations are warranted to explore the role of SHR in relation to adverse outcomes in stroke patients from diverse ethnic populations. Furthermore, there is a need to explore the potential benefits of stress hyperglycemia control in alleviating the physical health burdens associated with AIS. Maintaining a lower SHR level may potentially reduce the risk of adverse stroke outcomes.

## Data availability statement

The original contributions presented in the study are included in the article/Supplementary material, further inquiries can be directed to the corresponding authors.

## Author contributions

Y-WH conceptualized the study. Z-PL and X-SY developed and refined the search strategy. Y-WH and X-SY designed the study. Y-WH drafted the initial manuscript and Z-PL revised it. All authors have made significant contributions to this article and have given their full approval of the submitted version.
